# Supplementary material for: Identification and functional analysis of non-coding regulatory small RNA FenSr3 in Bacillus amyloliquefaciens LPB-18
Source: PeerJ. 2023 May 15;11:e15236. doi: 10.7717/peerj.15236 (PMC10194069; doi:10.7717/peerj.15236)
Supplement: Supplemental Information 4 [file peerj-11-15236-s004.zip › KO/CK-vs-T1_map/map04621.html]

KEGG PATHWAY: NOD-like receptor signaling pathway - Reference pathway


|  |  |
| --- | --- |
| **NOD-like receptor signaling pathway - Reference pathway** |  |

[
Pathway menu
| Organism menu
| Pathway entry
| Show description
| User data mapping
]

Specific families of pattern recognition receptors are responsible for detecting various pathogens and generating innate immune responses. The intracellular NOD-like receptor (NLR) family contains more than 20 members in mammals and plays a pivotal role in the recognition of intracellular ligands. NOD1 and NOD2, two prototypic NLRs, sense the cytosolic presence of the bacterial peptidoglycan fragments that escaped from endosomal compartments, driving the activation of NF-{kappa}B and MAPK, cytokine production and apoptosis. On the other hand, a different set of NLRs induces caspase-1 activation through the assembly of multiprotein complexes called inflammasomes. The activated of caspase-1 regulates maturation of the pro-inflammatory cytokines IL-1B, IL-18 and drives pyroptosis.

|  |  |  |
| --- | --- | --- |
| Reference pathway | 184% 150% 122% 100% 82% 67% 55% | 图片下载 |
